# Multi-Drug-Resistant Gram-Negative Microorganisms: Epidemiology, Treatment and Alternative Approach

**DOI:** 10.3390/antibiotics11050678

**Published:** 2022-05-18

**Authors:** Maria Teresa Mascellino

**Affiliations:** Department of Public Health and Infectious Diseases, Sapienza University of Rome, Piazzale Aldo Moro 5, 00185 Rome, Italy; mariateresa.mascellino@uniroma1.it; Tel.: +39-06499-70880

The presence of enzymes such as Extended-Spectrum β-lactamase (ESBL) and carbapenemases (KPCs, Metallo β-lactamases and OXA) constitutes the principal resistance mechanism to antibiotics. The spread of multi-drug-resistant organisms (MDROs), especially carbapenem-resistant (CR)- *Enterobacterales* (CRE) or, more correctly, carbapenem-producing *Enterobacterales* (CPE), has become a major problem worldwide with a high impact on both morbidity and mortality in humans. The recent approval of beta-lactam/beta-lactamase inhibitors (BLBLI) combination, such as ceftolozane-tazobactam, ceftazidime-avibactam, imipenem-relebactam, meropenem-vaborbactam, piperacillin-tazobactam, aztreonam-avibactam, offers solutions in the management of difficult-to-treat microorganisms, such as *Pseudomonas aeruginosa* and *Acinetobacter baumannii*, which continue to be a challenging problem for physicians due to several resistance mechanisms, which lead to limited treatment options. CRE have undergone extensive dissemination worldwide, resulting in a global threat to public health. Additionally, the predominant production of *Klebsiella pneumoniae* carbapenemase (KPC) contributes to the most important mechanism of carbapenem resistance. Carbapenemase gene detection is crucial to evaluate the molecular epidemiology, the risk factors and the outcome of MDROs infection. Furthermore, it would be desirable to look for any alternative approach, such as N-acetylcysteine or other compounds, which, combined with beta-lactams, may increase or restore their activity.

In this Special Issue (SI), different topics are taken into account in a thorough and accurate way, shedding light on this matter, which is currently crucial in the therapy of resistant infections.

An important topic in this collection of articles covers the use of antibiotics combination to fight the multi-drug resistance. The use of different dosages of cefoperazone–sulbactam (CFP–SUL g/1 g or 2 g/2 g every 12 h) for more than 1 week was tested by Chien-Hsiang Tai and co-workers in Taiwan in a population with impaired renal function presenting with septicemia caused by Gram-negative organisms [1]. The selected patients were divided in two groups belonging to various concentrations of antibiotics. No significant differences between the groups were found in all-cause mortality rates and adverse effects. The adverse effects of CFP–SUL did not increase in septic patients with renal insufficiency receiving CFP–SUL 2 g/2 g Q12H. However, the study could have been underpowered to identify a significant difference in all-cause mortality or other clinical outcomes. As a result of this, the authors are planning to look for additional in-depth studies concerning pharmacokinetic–pharmacodynamic optimization to verify the effects of different dosages of CFP–SUL on these kinds of patients.

For this purpose, the article by Yukihiro Hamada et al. related to the pharmacokinetic–pharmacodynamic (PK/PD) optimization of two antibiotics (cefmetazole and flomoxef) emerges as being very interesting [2]. These antimicrobials were examined with the aim to overcome the bacterial resistance and to establish a correct treatment in urinary tract infections caused by ESBL-producing *Enterobacterales*. An indicative bactericidal activity was detected for both antibiotics. The performance of cefmetazole and flomoxef in patients who received a standard regimen (0.5 or 1 g, 1 h injection) was calculated for each renal function. The optimal dosage was determined on the basis of creatinine clearance values, so that appropriate doses of these drugs were determined according to renal function. It can be inferred that the clinical definition of PK/PD plays a critical role in controlling antimicrobial resistance (AMR).

A very interesting manuscript concerning the use of antibiotics association is reported in the review by Belati A. et al. [3]. The meropenem/vaborbactam combination with aztreonam was evaluated as treatment strategy for the sepsis caused by ceftazidime/avibactam-resistant *Klebsiella pneumoniae* (CAZ/AVI-R-*Kp*). The meropenem/vaborbactam association plus aztreonam (even if it was discontinued) plays an important role in the therapy of MDR-Gram-negative bacteria, appearing efficacious against CRE with class A carbapenemases like KPC and class B metallo-betalactamase like NDM. Three patients with blood cultures positive for CAZ/AVI-R-*Kp* (one patient with *K. pneumoniae* KPC and two with *K. pneumoniae* NDM) were taken into account. Two patients recovered completely, whereas the third one with NDM-*Kp* died due to complications of the underlying disease, which required hospitalization. This association could be a valid option where the presence of OXA enzymes is rare. Unfortunately, the carbapenemase gene typing was not available, so the antibiotics susceptibility was only based on phenotypic results. 

This inconvenience was overcome in the research performed by Jia Jie Woon et al. in which the molecular characterization of carbapenem-resistant *Acinetobacter baumannii* (CRAB) was examined [4]. This microorganism is involved in severe infections, leading to complications that can become fatal, especially in immunocompromised patients. Colistin emerged as being active against CRAB. OXA carbapenemase gene was found to be the predominant one, with almost all the isolates co-harboring *blaOXA-23*-like and *blaOXA-51*-like genes. Different subtypes were isolated through PFGE. The authors found that age, infection by CRAB, ethnicity, co-morbidity and specimen source were significantly associated with a high rate of mortality. Appropriate measures, such as infection control, early identification of the sample source, targeted interventions in the management of the CRAB disease, could limit such infection in the ICUs and lower hospital mortality.

Considering that MDROs infection represents a considerable problem and that antimicrobial resistance remains one of the greatest public threats, alternative solutions were proposed to overcome this issue. Different approaches have been described, including compounds that appear efficacious against these infections. Two manuscripts in the SI address this problem. The first one by De Angelis M. et al. studied the activity of N- acetylcysteine (NAC) in combination with beta-lactams against carbapenem-resistant *Klebsiella pneumoniae* and *Acinetobacter baumannii*, whereas the second one by Meade E. et al. evaluated the effective antimicrobial solutions for eradicating multi-resistant pathogen bacteria [5,6]. In the first article, NAC, in addition to the beta-lactams (meropenem for CR-*Kp,* meropenem and ampicillin/sulbactam for CRAB, respectively), was not only able to enhance the carbapenems action but also to restore their activity. Moreover, the addition of NAC to meropenem or ampicillin/sulbactam at sub-inhibitory concentrations induced a fast and lasting bactericidal activity that persisted over time, causing a profound alteration of bacterial cell integrity. In the second manuscript, the use of biocides against isolated nosocomial pathogens (namely peracetic acid triameen and benzalkonium chloride) that act as disinfectants in the hospital environment and other therapeutic options, such as the use of phendione, which showed promising antimicrobial activity with low MIC values, were taken into account.

Another kind of resistance described in this SI concerns quinolone activity in *Salmonella* serovars, which is associated with mutations in the quinolone-resistance-determining regions (QRDRs), including DNA-gyrase and topoisomerase IV [7]. The position and type of the mutation (s) in the QRDRs made the bacteria partially or completely resistant to quinolones, The manuscript is a complete and detailed review of quinolone resistance in both typhoidal and non-typhoidal *Salmonella* serovars (especially *S. enterica*) due to plasmid-mediated quinolone-resistance determinants and to membrane quinolone efflux pumps in strains with QRDR mutations. The obtained results could be very useful for an accurate surveillance of foodborne *Salmonella* serovars with a high impact on food imports, which is the cause of the occurrence of non-typhoidal *Salmonella* in developed countries.

The impact of MDROs infection on patient length of stay (LOS) in respiratory care ward (RCW) was evaluated by Yi-Ping Chen and co-workers in Taiwan [8]. The LOS of patients in the RCW is an important parameter for evaluating the risk factors (such as catheterization, old age, immunodeficiency, hospital setting and previous use of antibiotics). In this clinical study, two groups were examined: the former included patients infected with multi-drug-resistant bacteria, whereas the latter (control group) included patients not infected with these microorganisms. The LOS of the subjects belonging to the control group was significantly lower than of those belonging to the first group with MDROs infection, and among these patients, carbapenem-resistant *Pseudomonas aeruginosa* was associated with a longer LOS than other multi-drug-resistant strains. The results of this study provided useful insights about the impact of MDROs on LOS of patients in respiratory wards.

In summary, MDR Gram-negative bacteria, such as *Enterobacterales*, *Acinetobacter baumannii* and *Pseudomonas aeruginosa*, represent a crucial problem in establishing a correct and appropriate therapy. Antibiotics association appears as a better solution than monotherapy with a lower mortality rate. The beta-lactam/beta-lactamase inhibitors combination, such as meropenem-vaborbactam, ceftolozane-tazobactam, ceftazidime-avibactam, cefoperazone-sulbactam, are thought to be a good solution, even if they sometimes fall short of expectations. Hence, it could be important to examine the innovative combinations of antibiotics, so that suitable treatments might be suggested. An alternative approach based on non-conventional antibiotic therapy, such as biocides, essential oils, bacteriophages, phendione and N-acetylcysteine, would be highly recommended. Currently, different plant-based inhibitors and synthetic peptides are being investigated for their capacity to fight drug resistance.
